# Dosimetric effects of positioning shifts using 6D‐frameless stereotactic Brainlab system in hypofractionated intracranial radiotherapy

**DOI:** 10.1120/jacmp.v17i1.5682

**Published:** 2016-01-08

**Authors:** Hosang Jin, Vance P. Keeling, Imad Ali, Salahuddin Ahmad

**Affiliations:** ^1^ Department of Radiation Oncology University of Oklahoma Health Sciences Center Oklahoma City OK USA

**Keywords:** frameless stereotactic radiotherapy, dosimetric effects, translational and rotational shifts, PTV margin, hypofractionation

## Abstract

Dosimetric consequences of positional shifts were studied using frameless Brainlab ExacTrac X‐ray system for hypofractionated (3 or 5 fractions) intracranial stereotactic radiotherapy (SRT). SRT treatments of 17 patients with metastatic intracranial tumors using the stereotactic system were retrospectively investigated. The treatments were simulated in a treatment planning system by modifying planning parameters with a matrix conversion technique based on positional shifts for initial infrared (IR)‐based setup (XC: X‐ray correction) and post‐correction (XV: X‐ray verification). The simulation was implemented with (a) 3D translational shifts only and (b) 6D translational and rotational shifts for dosimetric effects of angular correction. Mean translations and rotations (± 1 SD) of 77 fractions based on the initial IR setup (XC) were 0.51±0.86 mm (lateral), 0.30±1.55 mm (longitudinal), and −1.63±1.00 mm (vertical); 0.53±0.56 mm (pitch), 0.42±0.60 mm (roll), and 0.44±0.90 mm (yaw), respectively. These were −0.07±0.24 mm, −0.07±0.25 mm, 0.06±0.21 mm, 0.04±0.23 mm, 0.00±0.30 mm, and 0.02±0.22 mm, respectively, for the postcorrection (XV). Substantial degradation of the treatment plans was observed in $D_{95}$ of PTV (2.6%±3.3%; simulated treatment versus treatment planning), $D_{\text{min}}$ of PTV (13.4%±11.6%), and $D_{\text{min}}$ of CTV (2.8%±3.8%, with the maximum error of 10.0%) from XC, while dosimetrically negligible changes (< 0.1%) were detected for both CTV and PTV from XV simulation. 3D angular correction significantly improved CTV dose coverage when the total angular shifts (|pitch|+|roll|+|yaw|) were greater than 2°. With the 6D stereoscopic X‐ray verification imaging and frameless immobilization, submillimeter and subdegree accuracy is achieved with negligible dosimetric deviations. 3D angular correction is required when the angular deviation is substantial. A CTV‐to‐PTV safety margin of 2 mm is large enough to prevent deterioration of CTV coverage.

PACS number: 87.55.dk

## I. INTRODUCTION

Frameless stereotactic radiotherapy systems have played an important role in intracranial radiosurgery or radiotherapy allowing for noninvasive patient immobilization with a high precision of image‐guided (IG) positioning.^1^, ^2^, ^3^, ^4^, ^5^, ^6^ These systems allow patient setup with six dimensional (6D) corrections with three dimensional (3D) translational and 3D rotational shifts. One of the frameless stereotactic systems, Brainlab (Munich, Germany) ExacTrac X‐ray 6D system, employs stereoscopic X‐ray image‐guidance based on bony anatomy to calculate the translational and rotational shifts, which are then applied using a 6D robotic couch. Mechanical accuracy of the system has been extensively investigated.^7^, ^8^, ^9^, ^10^, ^11^, ^12^, ^13^, ^14^, ^15^, ^16^, ^17^ Ackerly et al.^(7)^ presented the overall spatial accuracy of the Brainlab ExacTrac system, which was 1.24 mm for stereotactic radiotherapy (SRT) and 1.35 mm for stereotactic radiosurgery (SRS) involving iterative 6D image fusion corrections. Lamba et al.^(9)^ reported that the frameless Brainlab system was comparable to the Brainlab frame‐based radiosurgery (the differences were 0.9±0.5 mm for anteroposterior, 0.2±0.4 mm for supero–inferior, and 0.3±0.5 mm for lateral, respectively). The ExacTrac X‐ray image‐guidance also showed a good agreement with cone‐beam computed tomography (CBCT)‐based registration (the root‐mean‐square of differences for translations was < 0.5 mm for phantom and < 1.5 mm for patients).^(10)^ Some research groups^11^, ^16^ showed that the 6D correction improved the target positioning better than the 3D translational method and the 4D positioning correction (three translations and the isocentric rotation). Several research efforts^14^, ^15^, ^17^ demonstrated that the ExacTrac positioning and frameless mask immobilization have submillimeter accuracy for intracranial tumors.

Although positioning setup errors using the Brainlab system have been well reported, dosimetric impact of these shifts for hypofractionated SRT has limitedly been investigated. The purpose of this study is four‐fold: 1) to investigate translational and rotational shifts of patient setup prior to irradiation using the Brainlab ExacTrac system for hypofractionated intracranial radiation therapy; 2) to quantify their dosimetric consequences using a novel matrix conversion of plan parameters; 3) to determine necessity of rotational correction; and 4) to evaluate a clinical target volume (CTV) to planning target volume (PTV) expansion margin to cover CTV with the setup errors.

## II. MATERIALS AND METHODS

### A. Patient selection and Brainlab setup verification

This retrospective study was performed involving 17 patients with metastatic intracranial tumors treated with the frameless stereotactic Brainlab ExacTrac system. The patients were treated in 3 or 5 fractions with prescription doses ranging from 20 to 40 Gy, as shown in Table 1. The original treatment plans were created with a Brainlab iPlan treatment planning system (TPS; version 4.1) to cover at least 95% of PTV by the prescription dose using 9 to 13 noncoplanar beams with sliding window intensity‐modulated radiation therapy (IMRT) technique. The treatments were delivered using a TrueBeam STx (Varian Medical Systems, Palo Alto, CA) equipped with high‐definition 120 multileaf collimator (HD 120 MLC) of 2.5 mm leaf width, the Brainlab ExacTrac image‐guidance system, and the 6D robotic couch. The patients were immobilized with a noninvasive rigid frameless mask. Then, they were simulated with a CT localization frame that was used to define an accurate stereotactic reference frame in the TPS. PTV was generated by expanding CTV with 1 to 3 mm safety margin (1 mm for one patient only [#9 in Table 1], 2 mm for 12 patients, and 3 mm for 4 patients) based on location of the intracranial lesions, proximity to critical structures, size of the lesions, and MRI‐CT image registration. They were mostly based on physicians' decision by mainly considering systematic errors or uncertainties in patient positioning. For example, if a lesion was next to a critical structure such as brainstem or chiasm, small margins were used. For large lesions, small margins were used in order to preserve normal tissues, while large margins were used for small lesion in order to ensure dose coverage. If contrast uptake was not clear on the MRI images with blurred boundaries, then the physicians decided to include larger margins. For lesions close to the skull and bone anatomy, smaller margins were used, while larger margins were used if targets were far from bone anatomy that was used for image‐guidance with the ExacTrac system. The shifts calculated with image registration of radiographs with DRRs for lesions (isocenter) close to bones are generally more accurate than those for lesions far away from bones because the ExacTrac system uses bone anatomy matching. When the isocenter is far from bones, the shifts produced by image registration are based on anatomical matching outside the region of interest, which is usually associated with large rotation shifts. The ExacTrac system was used to set up the patients in each treatment session. This system employed an infrared (IR) optical positioning system for initial patient setup based on the stereotactic reference frame using the treatment planning CT images. This was followed by stereoscopic kV X‐ray imaging for patient positioning based on bone anatomy matching where 3D translational and 3D rotational shifts were determined. These shifts were applied to correct patient position using the 6D robotic couch if the current position was out of our treatment tolerance of 0.7 mm and 1° in any direction or angle, respectively. Afterwards, a second set of X‐ray images were acquired to verify that the patient position was within treatment tolerance. Detailed descriptions of the system can be found in other publications.^9^, ^10^, ^11^

**Table 1:** Summary of patients selected for this study.

*Patient #*	*Treatment Site*	*PTV (cc)*	*Dose/Fraction (Gy)*	*# of Fractions*
1	Right temporal	37.2	8.0	3
2	Right cerebellum	58.4	5.0	5
3	Left maxillary sinus	19.9	8.0	5
4	Left frontal	93.9	6.0	5
5	Right temporal	9.6	5.0	5
6	Right frontal	26.2	7.0	5
7	Right optic track	32.9	4.0	5
8	Right cerebellum	21.5	5.0	5
9	Multiple targets	7.8	7.0	3
10	Left occipital	10.4	9.0	3
11	Optic chiasm	8.2	4.0	5
12	Left parietal	8.0	9.0	3
13	Right temporal	39.2	5.0	5
14	Left temporal	20.0	5.0	5
15	Left maxillary sinus	8.9	6.0	5
16	Right temporal	36.7	5.0	5
17	Brainstem	9.6	5.0	5

### B. Treatment simulation with the detected errors

Treatment plans were generated incorporating the translational and rotational shifts calculated by the ExacTrac system to simulate dose delivery without position corrections. The initial treatment plans were modified by newly computed machine parameters such as gantry, collimator, couch angles and isocenters, using a method proposed by Yue et al.^(18)^ In this method, the original treatment planning parameters are modified by a matrix conversion to produce a new combination of machine parameters and isocenters that simulates treatments with the 6D detected shifts. The planned dose data created in the iPlan TPS were transferred to a Varian Eclipse TPS (version 11.0) via DICOM format and recalculated with anisotropic analytical algorithm (AAA) and calculation grid size of 1 mm. The Eclipse TPS was selected as planning system because it has more capabilities to perform the modification of the planning parameters. For each patient, the new planning parameters for the simulated treatment were computed for each fraction with the corresponding shifts detected. A cumulative plan with dose summation of the entire three to five treatments was then generated. Two simulations of the treatment plans were created. First, plans were produced using 3D translational shifts only (referred to as “3D” hereinafter) that represent hypothetical treatments without rotational shifts. Second, plans with 6D translational and rotational shifts (referred to as “6D”) were simulated to investigate dosimetric consequences of angular corrections in comparison to 3D. A dose change (ΔD=(Planned dose‐Simulated dose)/(Planned dose)×100%) by the translational and rotational shifts was computed to evaluate dosimetric variations in CTV ($D_{99}$ (dose to 99% of volume), $D_{\text{min}}$ (minimum dose), $D_{\text{max}}$ (maximum dose), and $D_{\text{mean}}$ (mean dose)). The variation in dose coverage of PTV was evaluated using $D_{95}$ (dose to 95% of volume), $D_{\text{min}},\;D_{\text{max}}$, and $D_{\text{mean}}$. In critical organs such as brainstem, optic chiasm, and optic nerve, $D_{\text{max}}$ and $D_{\text{mean}}$ were evaluated. The translational and rotational shifts from two imaging stages were considered: 1) the initial IR setup verification (XC: X‐ray Correction as in the Brainlab ExacTrac positioning report), and 2) postcorrection (XV: X‐ray Verification). If the shifts from the initial XC for a fraction were within the tolerance level, they were also used for the XV simulation. If multiple X‐ray verifications were required due to large offsets by patient motion or relaxation, the last verification shifts right before treatments were used for the XV simulation. Several XV translational and rotational shifts were not further corrected for treatments, if they were slightly above tolerance level after multiple corrections (refer to the maximum and minimum values in Table 2). For a statistical significance test of comparison, a two‐tailed paired Student's *t*‐test was used at a significance level of 5%. A correlation was determined by the square of the Pearson product moment correlation coefficient $\left(r^{2}\right)$.

**Table 2:** Detected translational and rotational errors.

	*Translational (mm)*				*Rotational (°)*			
	*Lateral (x)*	*Longitudinal (y)*	*Vertical (z)*	$3D\;vector\;((\surd x^{2}+y^{2}+z^{2}))$	*Pitch* $\left(\theta_{x}\right)$	*Roll* $\left(\theta_{y}\right)$	*Yaw* $\left(\theta_{z}\right)$	*Angle summation* $\left(\left|\theta_{x}\right|+\left|\theta_{y}\right|+\left|\theta_{z}\right|\right)$
*X‐ray Correction (XC) – Initial Infrared‐based Setup*								
Mean^a^ (SD)	0.51 (0.86)	0.30 (1.55)	−1.63 (1.00)	2.45 (1.04)	−0.53 (0.56)	0.42 (0.60)	0.44 (0.90)	None
Maximum	3.71	4.33	0.95	5.26	1.20	2.60	2.80	5.30
Minimum	−1.88	−4.49	−4.20	0.32	−2.10	−1.40	−2.40	0.30
Mean Absolute^b^ (SD)	0.73 (0.68)	1.20 (1.02)	1.68 (0.90)	2.45 (1.04)	0.64 (0.47)	0.61 (0.51)	0.75 (0.77)	1.84 (1.07)
*X–ray Verification (XV) – Postcorrection*								
Mean^a^ (SD)	−0.07 (0.24)	−0.07 (0.25)	0.06 (0.21)	0.35 (0.22)	0.04 (0.23)	0.00 (0.30)	−0.02 (0.22)	None
Maximum	0.41	0.56	0.78	1.25	0.60	1.80	0.60	2.40
Minimum	−0.92	−0.64	−0.45	0.04	−0.70	−0.90	−0.90	0.0
Mean Absolute^b^ (SD)	0.19 (0.16)	0.19 (0.17)	0.16 (0.15)	0.35 (0.22)	0.17 (0.16)	0.16 (0.25)	0.16 (0.15)	0.49 (0.41)

^a^ Mean: mean value of actual error (Δd=(Planned value‐Simulated value)/(Planned value)×100%).

^b^ Mean Absolute: mean value of absolute error (|Δd|=(Planned value‐Simulated value)/(Planned value)×100%).

## III. RESULTS

The translational and rotational shifts from 77 fractions for XC and XV are summarized in Table 2. Mean translations and rotations (± SD) based on the initial IR setup (XC) were 0.51±0.86 mm, 0.30±1.55 mm, and −1.63±1.00 mm, respectively, for lateral (x), longitudinal (y), and vertical (z) directions; and 0.53±0.56 mm, 0.42±0.60 mm, and 0.44±0.90 mm, respectively, for pitch (θ_x_), roll (θ_y_), and yaw (θ_z_). For the postcorrection (XV), these were −0.07±0.24 mm, −0.07±0.25 mm, 0.06±0.21 mm, 0.04±0.23 mm, 0.00±0.30 mm, and 0.02±0.22 mm, respectively. Since the mean of the raw translational shifts does not represent the actual magnitude of the shifts for each patient, means of absolute values (|ΔD|) were provided as well. Large variations in the translational and rotational shifts were observed for XC (up to 5.26 mm in 3D vector and 2.8° in yaw), which was much larger than the 1–3 mm expansion margin. However, the submillimeter translational accuracy and subdegree angular variations were achieved for X V, which were within our PTV margins and patient setup tolerance.

**Figure 1:**
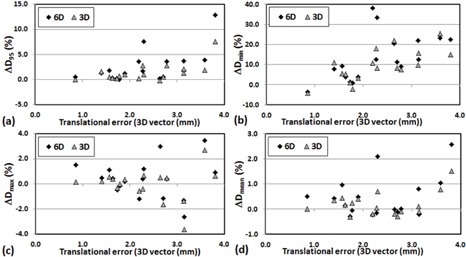
Change in PTV dose coverage ((Planned dose‐Simulated dose)/Planned dose×100%) with respect to the mean $3D\;\text{vector}(\surd (x^{2}+y^{2}+z^{2}))$ translational error of each patient for XC: (a) $\Delta D_{95}$, (b) $\Delta D_{\text{min}}$, (c) $\Delta D_{\text{max}}$, and (d) $\Delta D_{\text{mean}}$.

**Figure 2:**
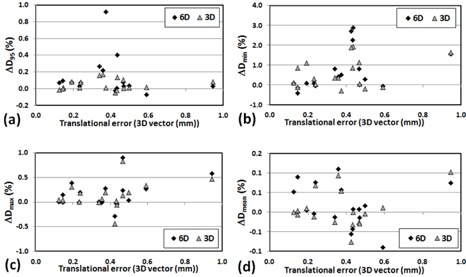
Change in PTV dose coverage ((Planned dose‐Simulated dose)/Planned dose× with respect to the mean 3D vector $3D\;\text{vector}(\surd (x^{2}+y^{2}+z^{2}))$ translational error of each patient for XV: (a) $\Delta D_{99}$, (b) $\Delta D_{\text{min}}$, (c) $\Delta D_{\text{max}}$, and (d) $\Delta D_{\text{mean}}$.

Table 3 shows the average change in CTV and PTV dose coverage (loss of coverage). Substantial degradation of the treatment plans was observed in $D_{95}$ of PTV, $D_{\text{min}}$ of PTV, and $D_{\text{min}}$ (the maximum error of 10.0%) of CTV for the XC simulation, which represented hypothetical treatments with the initial IR‐based setup only. However, dosimetrically negligible changes were calculated for both CTV and PTV of the XV simulation (< 1% for all; actual treatments). The shift corrections (difference between XC and XV) have significantly improved the dose coverage in $D_{95}$ (2.5% (p=0.008) in 6D and 1.4% (p=0.006) in 3D), and $D_{\text{min}}$ (12.7% (p=0.0002) in 6D and 8.9% (p=0.0002) in 3D) of PTV; however, there were no statistically significant changes in $D_{\text{max}}$ and $D_{\text{mean}}$ of PTV (*p* (p>0.05)). In general, a large translational error was translated to larger dosimetric change for $D_{95}$ and $D_{\text{min}}$ of PTV. Figure 1 shows strong correlation between positioning errors (mean of 3D translational shifts for each patient) and changes in $D_{95}(r^{2}=0.82)$ and $D_{95}(r^{2}=0.71)$ of PTV for 6D. However, the correlation diminished when the translational shifts were small (3D vector < 0.5 mm) for X V, as shown in Fig. 2. 3, 4 also show the dose changes in CTV for XC and XV, respectively. For CTV, changes in all of the dose coverage indices between XC and XV were not statistically significant, except for $D_{\text{min}}$ of CTV for 6D (p=0.02).

The effect of angular correction can be simulated by the difference between 6D ‐ XC and 3D ‐ XC assuming ideal matching after shift correction. The mean differences ((3D simulated dose‐6D simulated dose)/Planned dose) were 1.2%±3.9% for $D_{95}\;\left(p=0.04\right)$, 0.4%±0.3% for $D_{\text{min}}\;\left(p=0.10\right)$, 2.1%±9.3% for $D_{\text{max}}\;\left(p=0.03\right)$, and 0.7%±0.5% for $D_{\text{mean}}\;\left(p=0.03\right)$ of PTV; 0.3%±0.8% for $D_{99}\;\left(p=0.11\right)$, 1.6%±2.8% for $D_{\text{min}}\;\left(p=0.04\right)$, 0.1%±0.7% for $D_{\text{max}}\;\left(p=0.45\right)$, and 0.0%±0.3% for $D_{\text{mean}}\;\left(p=0.69\right)$ of CTV.

For the critical structures, the changes in dosimetric coverage strongly depended on proximity of the organs to PTV, degree of shift, and beam angle. For brainstem, the change in the 6D ‐ XC simulation ranged from −9.7% to 13.6% for $D_{\text{max}}$ and from −1.9% to 4.4% for $D_{\text{mean}}$. In the 6D ‐ XV simulation, the maximum change was 2.9% for $D_{\text{max}}$ and 0.3% for $D_{\text{mean}}$. For optic chiasm, all of the changes were less than 2% for both 6D ‐ XC and 6D – XV, except for three cases of 6D ‐ XC (up to 10.8% for $D_{\text{max}}$ and 6.8% for $D_{\text{mean}}$ of Patient #6 in Table 1) where the chiasm was abutting PTV (distance to PTV: 0.6 cm). For left and right optic nerves, the $D_{\text{mean}}$ and $D_{\text{max}}$ changes were less than 2.0% for all 6D and 3D simulations, except for four cases (Patients #3, #4, #6, and #16), in which the optic nerves were relatively close to the target (distance to PTV < 0.1 cm). The maximum dose escalations of the optic nerve were 10.0% ($D_{\text{mean}}$) and 27.6% ($D_{\text{max}}$) in the 6D ‐ XC simulation for Patient #6, whose distance to PTV was 2.8 mm. However, the changes were 0.4% ($D_{\text{mean}}$) and 2.7% ($D_{\text{max}}$) in the 6D ‐ XV simulation.

**Table 3:** Change (%) in target dose coverage ((Planned dose‐Simulated dose)/Planned dose×100%).

		*Target Coverage* ($\Delta D_{95}$ for PTV; $\Delta D_{99}$ *for CTV)*		$\Delta D_{\text{min}}$		$\Delta D_{\text{max}}$		$\Delta D_{\text{mean}}$	
		*6D*	*3D*	*6D*	*3D*	*6D*	*3D*	*6D*	*3D*
*X‐ray Correction (XC) – Initial Infrared‐based Setup*									
CTV	Mean	0.6	0.3	2.2	0.6	0.1	−0.1	−0.1	−0.1
	(SD)	(1.5)	(1.0)	(3.8)	(2.6)	(0.8)	(0.9)	(0.6)	(0.5)
PTV	Mean	2.6	1.4	13.4	9.5	0.3	−0.1	0.5	0.2
	(SD)	(3.3)	(1.8)	(11.6)	(8.0)	(1.5)	(1.3)	(0.8)	(0.5)
*X‐ray Verifcation (XV) – Postcorrection*									
CTV	Mean	−0.1	0.0	−0.1	0.0	0.0	0.1	0.0	0.0
	(SD)	(0.2)	(0.1)	(0.6)	(0.5)	(0.3)	(0.2)	(0.1)	(0.1)
PTV	Mean	0.1	0.0	0.7	0.6	0.2	0.1	0.0	0.0
	(SD)	(0.2)	(0.1)	(1.0)	(0.7)	(0.3)	(0.3)	(0.1)	(0.0)
*XC‐XV*									
CTV	Mean	0.7	0.3	2.2	0.6	0.0	−0.1	0.0	−0.1
	(SD)	(1.5)	(1.0)	(3.6)	(2.7)	(0.8)	(0.9)	(0.6)	(0.5)
PTV	Mean	2.5	1.4	12.7	8.9	0.2	−0.2	0.5	0.2
	(SD)	(3.4)	(1.8)	(11.2)	(7.6)	(1.6)	(1.4)	(1.8)	(0.5)

**Figure 3:**
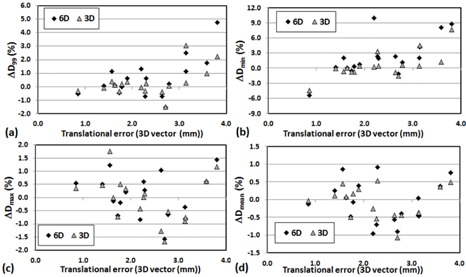
Change in CTV dose coverage ((Planned dose‐Simulated dose)/Planned dose× with respect to the mean 3D vector $3D\;\text{vector}(\surd (x^{2}+y^{2}+z^{2}))$ translational error of each patient for XC: (a) ΔD_99_, (b) $\Delta D_{\text{min}}$, (c) $\Delta D_{\text{max}}$, and (d) $\Delta D_{\text{mean}}$.

**Figure 4:**
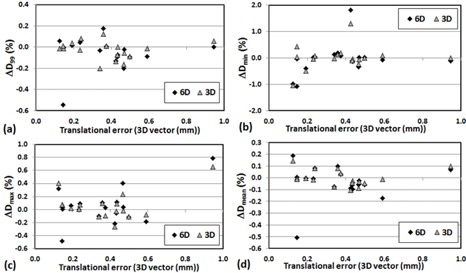
Change in CTV dose coverage ((Planned dose‐Simulated dose)/Planned dose× with respect to the mean 3D vector $3D\;\text{vector}(\surd (x^{2}+y^{2}+z^{2}))$ translational error of each patient for XV: (a) ΔD_99_, (b) $\Delta D_{\text{min}}$, (c) $\Delta D_{\text{max}}$, and (d) $\Delta D_{\text{mean}}$.

## IV. DISCUSSION

The initial IR‐based setup (XC) showed wide translational shifts ranging from −4.49 mm to 4.33 mm (the mean 3D setup error: 2.45±1.04 mm). Gevaert et al.^(16)^ similarly reported the mean 3D setup error before 6D correction was 1.91±1.25 mm and the rotational errors were 0.23±0.80 mm (pitch), 0.09±0.72 mm (roll), and 0.10±1.03 mm (yaw), respectively, for 40 patients immobilized with the Brainlab frameless mask. These errors were often much greater than our correction tolerance, which could produce clinically unacceptable dosimetric deviations. It indicates that the initial Brainlab positioning based on rigidity of the mask frame is not sufficient for accurate stereotactic positioning. Submillimeter accuracy was achieved with kV X‐ray verification and random errors (SD) were less than 0.3 mm (Table 2) using our Brainlab frameless stereotactic system. This is similar to the results by other research groups,^9^, ^11^, ^14^, ^17^ which also reported random errors of about 0.3 mm.

Treatments with XV shifts produced negligible deviation in CTV coverage (mean less than 0.40% with random errors less than 0.30% of all dosimetric indices) and most of the critical structures with 3D maximum shift of 1.25 mm and maximum angular shift $\left(\left|\theta_{x}\right|+\left|\theta_{y}\right|+\left|\theta_{z}\right|\right)$ of 2.40°. This supports that the 2–3 mm CTV‐to‐PTV margin can effectively cover CTV with the translational and rotational shifts detected. The large margin (3 mm) includes a lot of normal tissues that might be exposed by high doses and thus was limited to four cases when it was absolutely necessary in this study. We tried to customize the margins based on different factors, as previously explained, and ultimately based on consultation between physician and physicist. It should be noted that 2 mm margin was used for the majority of cases (12 out of 17) with one exception of 1 mm margin for a case with well‐defined, small targets. Verbakel et al.^(14)^ concluded that the SD of the X‐ray verification post‐RT could be used to calculate a margin for setup inaccuracy that was not different from the SD of the X‐ray verification prior to RT. Considering the SD of about 0.3 mm with 3D maximum shift of 1.25 mm and the uncorrected residual errors of up to translational 0.7 mm and rotational 1° of our Brainlab system, a minimum safety margin of 2.0 mm is required, which concurs with the previous report.^(13)^ However, lack of dose coverage up to 3% was found in $D_{\text{min}}$ of PTV (Patients #12 and #13) even with the XV submillimeter shifts. This is attributed to a few voxels at the periphery of PTV that are close to high‐dose gradients in conformal plans. Furthermore, inhomogeneity of PTV, which sometimes includes an air cavity or a bone as part of volume from expansion of CTV, may affect the dose coverage from modified plans with the small XV shifts.

The 3D translational shifts acquired in the 6D corrections might not be the best fits for the 3D setup correction. This study was a retrospective study that used acquired data, and unfortunately our current Brainlab system does not allow us to register the acquired images using 3D translational shifts only. In this study, the 3D translational shifts acquired for 6D correction were used for the 3D study to quantify the dosimetric deviations induced by the rotational shifts only using the same translational errors. Even if the 3D translational shifts from 6D corrections might not be the best, the 3D XV simulations showed the changes in the target coverage ($D_{95}$ of PTV and $D_{99}$ of CTV) less than 0.2% for all of the cases, indicating negligible errors induced by the 3D translational shifts which might be close to the best shifts. However, there were several outliers in $D_{95}$ of PTV (1 out of 18 cases greater than 3% change) and $D_{99}$ of CTV (3 out of 18 cases greater than 1% change) in 3D XC simulations. These cases can be improved using a better 3D registration without considering angular corrections.

Dosimetric consequences of translational and rotational shifts are usually simulated in a TPS by two main methods. In this study, the planning parameters were modified without changing the original CT data set. Kim et al.^(19)^ showed effectiveness of this method to evaluate dosimetric impact of setup positioning errors for spine stereotactic body radiotherapy. This approach is not affected by any uncertainties of manipulation of CT image, but mainly restricted by intrinsic limitation of a planning system that sometimes does not allow planners to modify vital planning parameters. The second approach uses the planning CT dataset translated and rotated with detected shifts that is inherently affected by accuracy of image transformation. Guckenberger et al.^(20)^ exploited this method to investigate dosimetric consequences of 3D and 6D errors in cone‐beam CT‐based frameless image‐guided radiosurgery (IG‐RS). They detected 3.9±1.7 mm of 3D error and the maximum rotational error of 1.7±0.8 mm on average prior to IG correction, and 0.9±0.6 mm of post‐treatment 3D error for 98 brain metastases, and concluded that each 3D setup error of 1 mm decreased target coverage by 6% with large interpatient variability. Gevaert et al.^(16)^ also rotated the CT scans including structures (i.e., contours of target volume and organs at risk) with the Brainlab BrainScan TPS to evaluate a possible gain in 6D patient positioning and found that the mean conformity index (CI; a value close to 1 indicates the ideal conformation) increased from 0.59±0.12 (4D correction) to 0.68±0.08 (6D correction).

**Figure 5:**
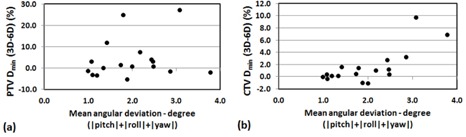
Effect of angular correction by $D_{\text{min}}$ coverage change of (a) PTV and (b) CTV with respect to magnitude of angular deviation using XC simulation. The positive value means the 6D translational and rotational correction can improve the $D_{\text{min}}$ coverage more than 3D translational correction only does.

For most of the cases, the 3D rotational shifts did not make substantial changes in terms of CTV and PTV coverage, as reported by Guckenberger et al.^(20)^ They demonstrated that pretreatment correction of translation mostly affected target coverage and conformity. However, remarkable differences between 6D and 3D were observed in $D_{\text{min}}$ when the large angular shifts were involved, as shown in Fig. 5, which resulted in target underdosing. If the mean value of absolute angular deviation $\left(\left|\theta_{x}\right|+\left|\theta_{y}\right|+\left|\theta_{z}\right|\right)$ of 3 or 5 fractions is greater than 2°, the 6D correction greatly improves the CTV $D_{\text{min}}$ compared to 3D translational correction only. This demonstrated the necessity of 3D angular correction when the angular shifts are greater than a certain level (greater than 2° in this study). Our results are consistent with another study by Peng et al.,^(21)^ which demonstrated that with a CTV‐to‐PTV margin of 3 mm, rotational setup errors of 3° or less did not decrease CTV coverage to less than 95% for 10 stereotactic intracranial patients. It should be noted that the 3D angular correction is a complex interplay of magnitude of translational and rotational shifts, depending on the location of isocenter and PTV size and shape.

In the hypofractionated SRT (3 or 5 fractions), even if a fraction of a treatment was close to (or exceeded) the setup tolerance of 0.7 mm, the average 3D translation errors after XV for most of the patients was less than 0.3 mm. This implies that the dosimetric errors by spatial offsets can be alleviated by multiple fractions in hypofractioned SRT. The same tolerance of 0.7 mm and 1° can make a larger impact on target coverage and surrounding health tissues in single‐fraction SRS, especially when critical structures are adjacent to CTV. A smaller safety margin, tighter setup tolerance, and verification images during treatment and repositioning using ExacTrac might be required for SRS that can be evaluated by the method used in this study.

This study was limited because it did not take into account added uncertainty due to couch rotation. The X‐ray verification was performed at the couch angle of 0°. The detected shifts were propagated through entire treatment; however, shifts will be different if imaging is taken for noncoplanar beams. Based on our clinical observation, it might lead to setup uncertainty by up to 0.7 mm translational shifts and 0.5° of rotational shifts from the 0° couch position. Another issue is intrafractional movement of patient during the treatments. Verbakel et al.^(14)^ reported that the Brainlab stereotactic system has the intrafractional shifts of −0.06±0.19 mm, −0.01±0.27 mm, and −0.04±0.23 mm, respectively, for vertical, longitudinal, and lateral directions. In another study,^(16)^ the mean 3D intrafraction shift was 0.58 mm (SD: 0.42 mm) for 43 patients undergoing hypofractionated SRT. Since the intrafractional shifts are relatively small and similar to our XV shifts, these will not substantially change the target coverage; however, a further study needs to be performed with outliers of intrafractional shifts. Verification images during treatment will contribute to alleviating the dosimetric change by intrafraction motion.

## V. CONCLUSIONS

The initial IR‐based setup in the frameless Brainlab ExacTrac system is not sufficient for accurate stereotactic positioning. With the 6D stereoscopic X‐ray verification imaging, submillimeter and subdegree accuracy is achieved with negligible dosimetric deviations in target coverage (< 1% compared to treatment planning) in hypofractionated SRT. 3D angular correction is required especially when the angular deviation is substantial (greater than 2° $\left(\left|\theta_{x}\right|+\left|\theta_{y}\right|+\left|\theta_{z}\right|\right)$). A CTV‐to‐PTV safety margin of 2 mm is large enough to prevent deterioration of CTV coverage with uncorrected residual errors of up to translational 0.7 mm (each direction) and rotational 1°. This study introduced a novel approach to investigate dosimetric deviation due to translational, rotational, and combined 6D shifts. The dosimetric evaluation was performed with matrix conversion of plan parameters at two patient setup stages that include X‐ray correction and verification. The dose evaluation using the matrix conversion was a useful tool to quantify dosimetric change of target coverage by positioning shifts in the frameless stereotactic Brainlab system.
